# Supplementary material for: Microbiota-Derived β-Amyloid-like Peptides Trigger Alzheimer’s Disease-Related Pathways in the SH-SY5Y Neural Cell Line
Source: Nutrients. 2021 Oct 29;13(11):3868. doi: 10.3390/nu13113868 (PMC8624230; doi:10.3390/nu13113868)
Supplement: Supplementary file 1 [file nutrients-13-03868-s001.zip › nutrients-1360427-supplementary.pptx]

## Slide 1
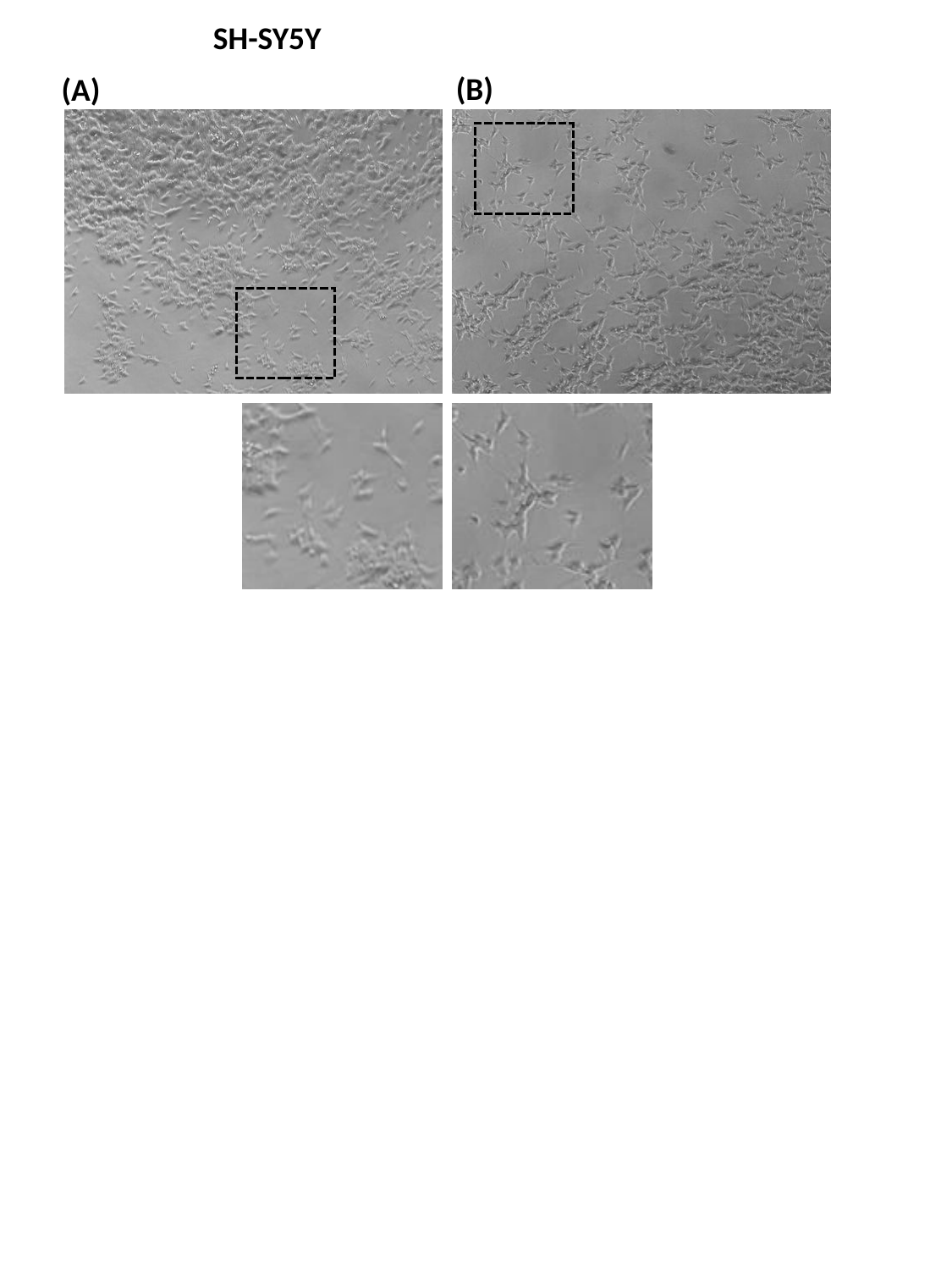

SH-SY5Y
(B)
(A)

## Slide 2
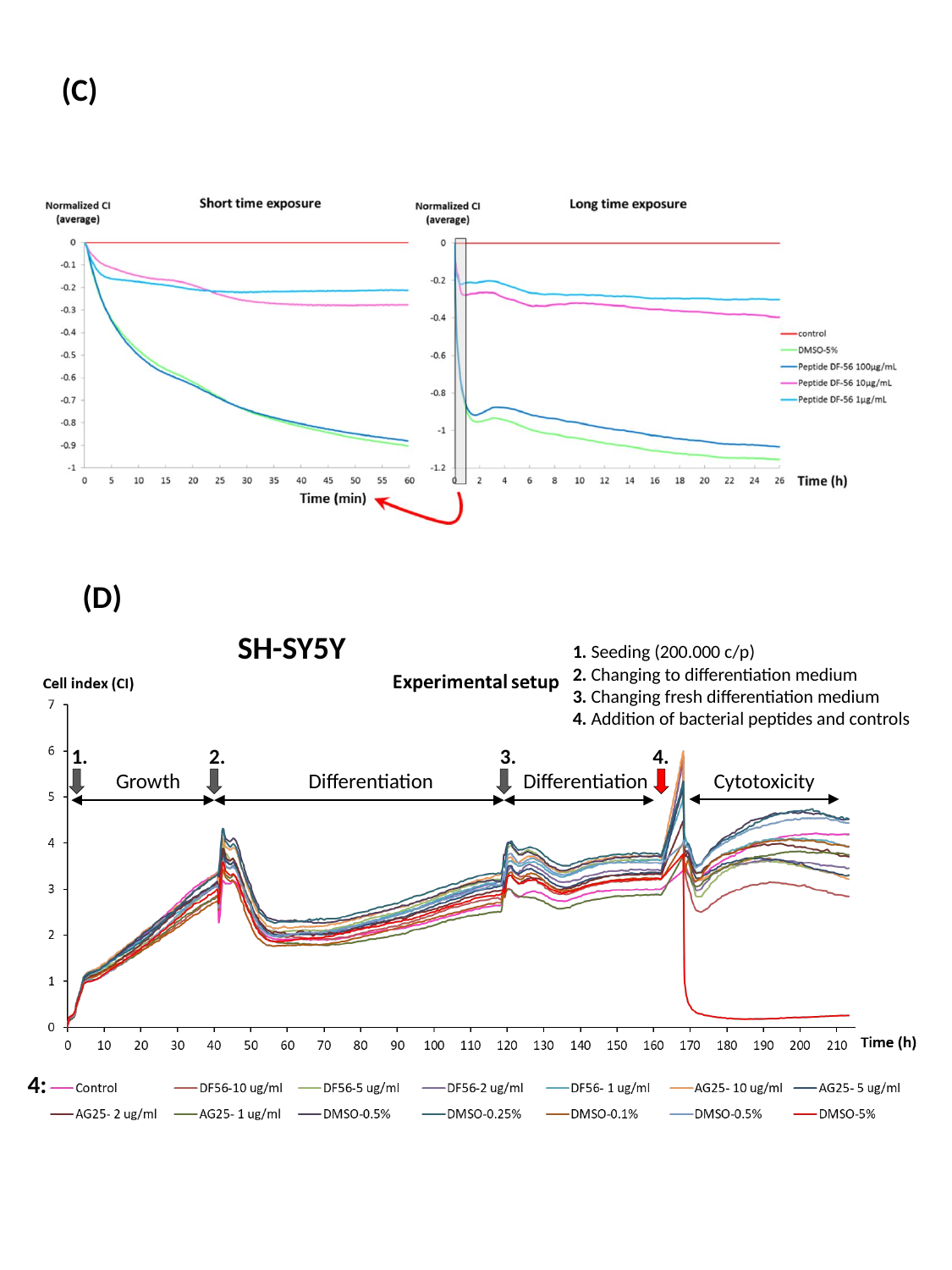

(C)
(D)
SH-SY5Y
1. Seeding (200.000 c/p)
2. Changing to differentiation medium
3. Changing fresh differentiation medium
4. Addition of bacterial peptides and controls
1.
2.
3.
4.
Growth
Differentiation
Differentiation
Cytotoxicity
4:

## Slide 3
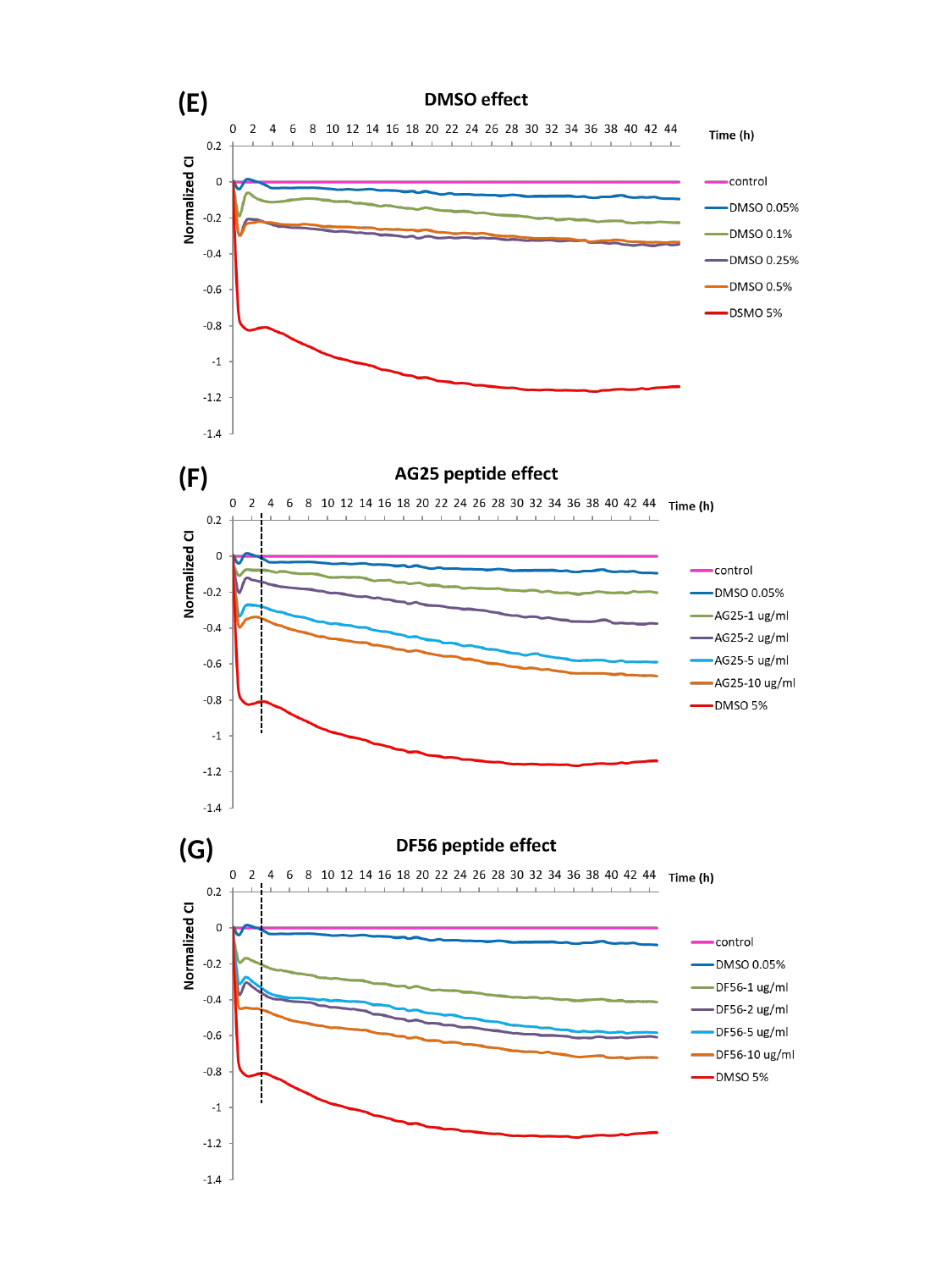

(E)
(F)
(G)
